# Proposing a Questionnaire for Assessing English Proficiency Among Japanese Medical Students: Current Perspectives and a Pilot Survey

**DOI:** 10.7759/cureus.44981

**Published:** 2023-09-10

**Authors:** Mason AlNouri, Keiichiro Maniwa, Toru Asari, Yasuyuki Ishibashi

**Affiliations:** 1 Department of Orthopedic Surgery, Hirosaki University Graduate School of Medicine, Hirosaki, JPN

**Keywords:** questionnaire, medical students, proficiency, language, medical english

## Abstract

Background

Japanese medical schools have made advances in terms of English and Medical English teaching in the past decade, in keeping with their importance in medical communication and research. English skills and proficiency levels differ across different institutions due to the variable adoption of general teaching requirements. A limitation in assessing English proficiency among Japanese medical students continues to exist due to the lack of standardized testing requirements.

Methods

A new questionnaire was developed by faculty members and medical students. Areas of importance were: demographics, proficiency and confidence ratings, history of learning and usage, duration of usage, perceived importance, and improvement goals. The final draft of the constructed questionnaire contained 21 questions in total. The questionnaire was administered over a three-month period in incremental order of enrollment through a digital online platform.

Results

A total of 133 students, 64 (48.1%) males and 69 (51.9%) females, participated. The average age was 23.7 ± 4.8 years. Based on an incremental Likert scale, respondents rated themselves as 1.0 ± 0.8 for English proficiency and 0.5 ± 0.7 for Medical English proficiency. The confidence level for English medical discussions was 0.2 ± 0.6 on a similar scale. Students on average attended 18.0 ± 30.0 classes per year and presented medical materials in English around 1.7 ± 1.7 times in total. The English language was used for 2.1 ± 6.3 hours per week in personal settings and 0.5 ± 1.7 hours per week in professional settings.

Conclusions

The proposed questionnaire was able to give valuable information about language skills and proficiency levels, but would require an incentive for improved participation. The pilot analysis showed that English and Medical English proficiency levels remain low with limited opportunities for using English in some areas within Japan. It may be beneficial to provide Japanese medical students with more occasions where they can use or practice their English skills.

## Introduction

The importance of English language proficiency in medical practice and training is undeniable in the modern era, owing to the widespread usage of English as a means of communication in many specialties [1-3]. Numerous examples can be given on how this affects medical literature and clinical outcomes, for instance: The higher citation rate of research articles published in English versus other languages [4], the exclusion of non-English articles by at least a third of systematic reviews [5], the increasing number of English publications versus those in other languages [6], the overwhelming dominance of English journals and articles in major indexing databases like PubMed/MEDLINE [7], the correlation of poor English skills with adverse clinical outcomes in some cases [8], and the list goes on. Although English takes first place as the most commonly used language around the world, non-native English speakers far outnumber natives, and there is a greater proportion of non-speakers in the global population [9]. Despite these facts, however, English remains the lingua franca of modern medicine [3].

Many traditionally non-English-speaking countries, where medicine is taught in the local language, have had to initiate policies and come up with strategies that enable medical students, trainees, and graduates to gain a basic understanding of the English language in the context of medical practice, colloquially termed “Medical English”. Japan is one of these countries that has worked on reforming teaching practices at medical schools in order to incorporate Medical English into their curriculums, in line with what is being taught in other countries [10]. This initiative was spearheaded by the Japan Society for Medical Education (JSME) in 2013 and furthered by the Japan Society for Medical English Education (JASMEE) in 2015, which published guidelines corresponding to the global standards for medical education by listing the minimum Medical English learning goals that should be achieved by Japanese medical students [11,12].

Requirements for Medical English in Japan

According to the JASMEE guidelines, medical students should meet defined basic targets in vocabulary, reading, writing, and communication skills for Medical English, by their graduation date in the sixth year of medical school [12]. A few examples are understanding basic terms, being able to search or look up simple topics, having a basic technical writing ability, being able to conduct a basic medical interview with a patient, and possessing the ability to present and answer questions about a simple medical or research topic [12]. If students wish to go a step further for personal or professional reasons, they may aim for some of the advanced targets listed alongside the minimum requirements. A few of the more difficult skills include having a thorough understanding of expressions and terminology, displaying a solid ability to discuss clinical and research topics, and being able to write medical reports and articles without difficulty [12]. The committee responsible for creating the guidelines had hoped to see improvements in Medical English among students and doctors-in-training, ultimately leading to a positive impact on the worldwide reputation of medical education in Japan [12].

While the JASMEE has worked hard on supporting different educational programs for the advancement of Medical English at various medical schools, it unfortunately does not possess regulatory powers. The Ministry of Education, Culture, Sports, Science and Technology (MEXT) and the Ministry of Health, Labour and Welfare continue to hold authority over curriculums before and after graduation, respectively [13]. As such, the JASMEE has limited powers when it comes to structural changes and enforcement, thereby giving rise to disparate levels of implementation at medical schools in Japan. This also occurs secondary to the dissimilar requirements for Medical English by the JASMEE, MEXT, and the Ministry of Health, Labour and Welfare. In the translated core curriculum published by the MEXT, English was only explicitly mentioned in the context of contributing to medical care internationally, without specifying a required level of competency [14]. Similarly, the Ministry of Health, Labour and Welfare does not discuss Medical English or related proficiencies within the Medical Practitioners’ Act [15].

Medical English at Japanese medical schools

Following the establishment of the JASMEE in July of 1998, surveys were conducted at 80 different medical schools to determine the general trends for Medical English education at the time [16]. The results were published in the first volume of JASMEE’s official journal, showing that classes dedicated to Medical English were on a decline partly due to a cramped schedule for students, especially in the initial year. The development of systems and services that support Medical English education has nonetheless been rising among Japanese medical schools in the 21st century. Some of the pioneers in these endeavors are Tokyo Medical University and the University of Tsukuba, which both possess centers focused on providing several teaching and editorial options for medical students and faculty members [10]. While it would be difficult to list initiatives by all the medical schools in Japan, a few notable examples can be mentioned: the establishment of a regular Medical English class for first-year students at Toho University [17], the creation of a similar class for junior students at Akita University [18], the expansion of Medical English programs at Miyazaki University [19], and the extension of Medical English classes to senior students at Tokyo Women’s Medical University [20].

Given the lack of unified requirements for Medical English proficiency, Japanese medical schools are free to choose the extent of English incorporation into their clinical curriculums. Unfortunately, this leads to differences in the levels of English and Medical English proficiency among medical students across Japan [21]. Medical schools in Japan may also have dissimilar incentives towards adopting more robust English classes, in relation to some variables like: ranking, prestige, cost, feasibility, and others. Additionally, private or public governance would also play a role in determining the extent of English teaching at medical schools. While it would be unrealistic to expect the same level of language education nationwide, there is an undeniable need to assess the current level of teaching at medical schools in order to ensure that they are capable of enabling medical students in Japan to achieve a basic minimum level of Medical English proficiency, needed to support their continued education, specialization, and career goals.

Assessing Medical English proficiency

Theoretically, language proficiency can be rated from the internal perspective of the student or the external perspective of a rater. Despite their problems, standardized English tests remain the de facto gold standard for identifying levels of proficiency in many regions using the external perspective of a well-established examination or scale [22]. These assessments are rather common in the context of clinical licensing and practice, where countries like the United States, United Kingdom, Canada, Australia, and New Zealand subject international graduates to rigorous standards in order to ensure an adequate level of English language proficiency prior to qualification approval. In Japan, as well as in other non-English-speaking countries, standardized language examinations are usually available to students but, in most cases, are not required by local universities. Consequently, Japanese medical students do not routinely sit for these assessments. Japanese medical schools usually administer written entry examinations, which generally include only listening and reading components in English, in line with what is taught at the secondary educational levels in public schools. This precludes the use of standardized assessment scores to determine English proficiency among medical students in Japan.

Until radical English education policy changes occur at schools and universities, identification of language proficiency on a wide scale in Japan is only realistically feasible from an internal perspective, where students rate their own skills using simple ordinal scales in a questionnaire format. This was employed in the past to determine general trends and opinions of medical students, but only on a basic and localized level [21]. Ideally, a unified Japanese questionnaire should be designed, validated, and distributed to representative medical schools in different regions within Japan. This would allow for some kind of standardization in identifying and comparing Medical English proficiency across the country. Furthermore, having a common questionnaire would raise awareness and encourage improvements in English language education at Japanese medical schools. Nationally administered surveys and questionnaires have been previously utilized in medical schools and were able to showcase important results that improved teaching in the long term [23]. The primary goal of this study was to create and pilot test an English proficiency questionnaire for Japanese medical students. The secondary goal was to determine and comment on current levels of English proficiency at the participating institution.

## Materials and methods

Questionnaire development

**Figure 1:**
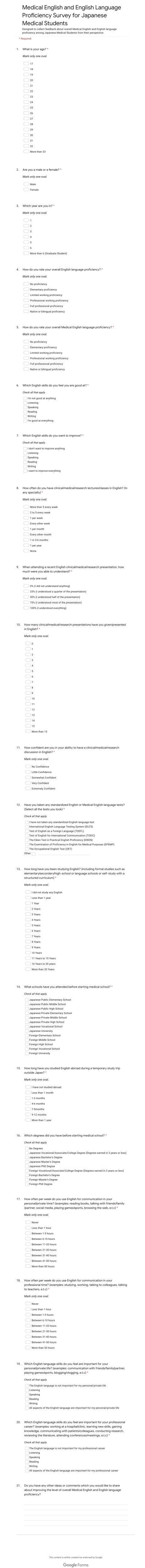
Medical English and English language proficiency survey for Japanese medical students.

**Figure 2:**
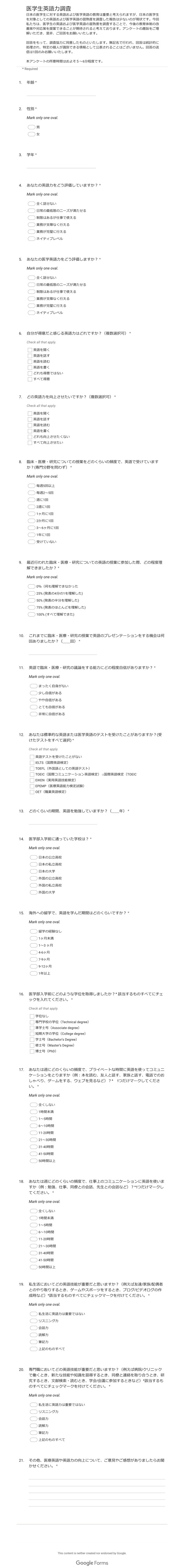
Medical English and English Language proficiency survey for Japanese medical students (Japanese).

A mixed-type questionnaire was developed through multiple rounds of extensive discussions with various faculty members and medical students. Areas of importance were determined to be: demographics, proficiency and confidence ratings, history of learning and usage, duration of usage, perceived importance, and improvement goals. Several questions were formed to ensure a complete assessment of all areas without overburdening the participants. A draft of the questionnaire was shared with a member of the JASMEE board of directors and the chief editor of the Journal of Medical English Education, who provided constructive comments and suggestions about the content of the questionnaire and areas of potential improvement. Following six rounds of revisions, the final draft of the proficiency questionnaire was constructed in English and Japanese, and contained 21 questions in total, which could be completed in about five minutes. Although only the Japanese version was distributed to medical students, both languages are included in the Appendix (Figures 1, 2) for the purpose of facilitating future validation and adaptation.

Pilot survey

A pilot survey involving the questionnaire was necessary in order to test its initial feasibility and effectiveness at a Japanese medical school while setting up the groundwork for validation. We consulted the institutional ethics committee regarding approval of the study and were advised that it was unnecessary since the research topic did not fall under institutional regulations on biomedical research and did not require ethics committee approval. Over a three-month period, the questionnaire was administered at Hirosaki University School of Medicine in incremental order of enrollment through a digital online platform (Google Forms), starting with junior-year medical students and ending with seniors. Students were given three weeks to respond, with weekly reminders. Confidentiality was maintained throughout the process by utilization of the online system, where students can answer the questions privately and anonymously through the same link to the questionnaire. There was no collection of any personal information beyond demographic data, mainly age and sex. Data was collected and stored within the Google Forms framework and server for analysis at a later stage. No incentives were provided for answering the questionnaire and participating in the study. Data analysis was performed using Microsoft Excel 2016 MSO Version 2203 Build 16.0.15028.20152 (Microsoft, Redmond, Washington). Results were aggregated by enrollment year and sex, with reporting of continuous variables as mean (standard deviation (SD)) and categorical variables as frequency distributions with percentages.

## Results

A total of 133 students, 64 (48.1%) males and 69 (51.9%) females, responded to the questionnaire. This number was 16.3% of the students at the medical school, who totaled 817 from all enrollment years. The average age of all participants was 23.7 ± 4.8 years. Table 1 shows stratified enrollment and participation statistics. On an incremental Likert scale of English proficiency, where 0 corresponds to “no proficiency” and 5 represents “native proficiency”, respondents rated themselves as 1.0 ± 0.8 for English proficiency and 0.5 ± 0.7 for Medical English proficiency. For lectures or sessions conducted entirely in English, students understood approximately 39.5 ± 27.7% of the content. In terms of engaging in English discussions of a clinical or medical nature, on an incremental Likert scale going from 0 “no confidence” to 4 “extremely confident”, their confidence level was rated as 0.2 ± 0.6. Regarding specific language skills, students most commonly thought they were good at reading (54.9%) and listening (31.6%), while 27.1% believed that they were not good at any skill. Writing (10.5%) and speaking (8.3%) skills were less commonly selected, and only 1.5% believed they were well-rounded in all English skills.

**Table 1:** Statistics for enrollment and participation stratified by year and sex. n = number; % = percent; yr = years.

	Enrolled			Responded			
Group	Students (n)	Males (n)	Females (n)	Students (n)	Age (yr)	Males (n)	Females (n)
All	817	472 (57.8%)	345 (42.2%)	133 (16.3%)	23.7 (4.8)	64 (48.1%)	69 (51.9%)
First year	117	75 (64.1%)	42 (35.9%)	19 (16.2%)	19.6 (1.9)	13 (68.4%)	6 (31.6%)
Second year	139	84 (60.4%)	55 (39.6%)	9 (6.5%)	20.7 (1.4)	2 (22.2%)	7 (77.8%)
Third year	130	67 (51.5%)	63 (48.5%)	36 (27.7%)	23.5 (4.2)	15 (41.7%)	21 (58.3%)
Fourth year	153	81 (52.9%)	72 (47.1%)	37 (24.2%)	24.7 (5.5)	14 (37.8%)	23 (62.2%)
Fifth year	133	71 (53.4%)	62 (46.6%)	24 (18%)	26.2 (5.2)	14 (58.3%)	10 (41.7%)
Sixth year	145	94 (64.8%)	51 (35.2%)	8 (5.5%)	25.3 (1.7)	6 (75%)	2 (25%)
Males	472	472 (100%)	0 (0%)	64 (13.6%)	23.8 (4.6)	64 (100%)	0 (0%)
Females	345	0 (0%)	345 (100%)	69 (20%)	23.6 (5.1)	0 (0%)	69 (100%)

In a single academic year, students on average attended 18.0 ± 30.0 classes. Junior year respondents had reported attending more clinical classes in English than their senior counterparts. Participants reported having the chance to present medical materials in English around 1.7 ± 1.7 times in total. For standardized English language proficiency tests, 38.3% of medical students sat for the EIKEN (Test in Practical English Proficiency) and TOEIC (Test of English for International Communication), 32.3% had not undertaken any test, 21.1% took the TOEFL (Test of English as a Foreign Language), and 6% sat for the IELTS (International English Language Testing System). Regarding school attendance prior to medical college enrollment, the majority of students reported attending a Japanese public school (67.7%). The remaining students attended a Japanese private school (16.5%), a Japanese university (14.3%), and a foreign university (1.5%). Therefore, only 1.5% of students possessed a master’s degree and 15% had a bachelor’s degree. The majority had not obtained any degrees (83.5%) beyond the high school diploma.

As for the duration spent on studying English during their lifetime, respondents averaged 3.7 ± 5.7 years. Moreover, students studied English abroad (outside Japan) for an average of 1.5 ± 4.9 months. Regarding their usage of English, 2.1 ± 6.3 hours per week went towards use in personal settings, while 0.5 ± 1.7 hours per week was the rate of use in professional settings. The importance of specific English language skills in a personal setting was reported by the following percentage of participants: 25.6% (none), 12.8% (listening), 6% (reading), 28.6% (speaking), 0% (writing), and 27.1% (all). For a professional setting, this was noted to be: 4.5% (none), 11.3% (listening), 9.8% (reading), 21.8% (speaking), 3.8% (writing), and 48.9% (all). All students wanted to improve upon their current levels of English and Medical English proficiency, specifically in the following skills: listening (27.1%), reading (9%), speaking (51.9%), writing (15%), and everything (41.4%). Most respondents had no additional comments (44.4%) regarding how to improve their level of English proficiency. The remaining participants hoped to see improvements in English classes (15%) and more opportunities for English usage (17.3%). Survey results for questions with numerical responses can be seen in Table 2. Non-numerical response percentages are shown in Table 3.

**Table 2:** Survey results for continuous numerical variables stratified by year and sex. /yr = per year; % = percent; yr = years; mo = months; hr/wk = hours per week.

Group	Personal English proficiency rating	Medical English proficiency rating	Clinical English classes (/yr)	Understanding in recent lecture (%)	Times presented	Confidence in Medical English discussions	Studying English duration (yr)	Lifetime study abroad (mo)	Private time English (hr/wk)	Work time English (hr/wk)
All	1.0 (0.8)	0.5 (0.7)	18.0 (30.0)	39.5 (27.7)	1.7 (1.7)	0.2 (0.6)	3.7 (5.7)	1.5 (4.9)	2.1 (6.3)	0.5 (1.7)
First year	1.3 (1.0)	0.7 (1.1)	55.5 (31.0)	54.0 (31.5)	0.2 (0.5)	0.1 (0.3)	8.4 (4.2)	2.7 (7.5)	0.9 (1.8)	1.1 (2.1)
Second year	0.8 (0.7)	0.3 (0.5)	6.9 (17.0)	41.7 (28.0)	1.8 (1.4)	0.2 (0.7)	9.2 (3.7)	3.4 (8.0)	0.2 (0.4)	0.1 (0.3)
Third year	0.9 (0.8)	0.3 (0.5)	24.8 (30.8)	30.6 (22.5)	1.6 (1.6)	0.1 (0.4)	2.6 (6.3)	1.1 (4.0)	2.2 (7.0)	0.4 (1.7)
Fourth year	0.9 (0.8)	0.6 (0.6)	2.6 (8.8)	38.5 (29.8)	1.6 (0.8)	0.4 (0.6)	2.7 (5.5)	0.6 (2.2)	3.2 (8.9)	0.4 (1.8)
Fifth year	1.1 (0.8)	0.5 (0.6)	11.8 (30.3)	44.8 (27.6)	2.4 (1.5)	0.5 (0.9)	2.1 (4.4)	2.1 (5.7)	2.1 (4.5)	0.6 (2.0)
Sixth year	0.4 (0.5)	0.3 (0.5)	0.5 (1.1)	31.3 (17.7)	3.3 (3.2)	0 (0)	0.5 (1.1)	0 (0)	1.4 (3.5)	0 (0)
Males	1.0 (0.7)	0.5 (0.7)	20.6 (29.0)	39.4 (27.8)	1.6 (1.6)	0.3 (0.7)	3.8 (5.9)	1.2 (4.0)	1.1 (2.6)	0.6 (2.0)
Females	1.0 (0.9)	0.5 (0.6)	15.6 (30.8)	39.5 (27.9)	1.7 (1.5)	0.2 (0.5)	3.6 (5.6)	1.7 (5.6)	3.0 (8.3)	0.3 (1.5)

**Table 3:** Survey results for non-numerical variables stratified by year and sex. % = percent, EPEMP: examination of proficiency in English for medical purposes, OET: occupational English test.

Group	Skills good at	Want to improve	English tests performed	Schools attended before med school	Degrees earned before med school	Skills important for private life	Skills important for work and career	Comments
All	Nothing: 36 (27.1%)	Nothing: 0 (0%)	Nothing: 43 (32.3%)	Japanese Public School: 90 (67.7%)	None: 111 (83.5%)	Nothing: 34 (25.6%)	Nothing: 6 (4.5%)	No comments: 59 (44.4%)
	Listening: 42 (31.6%)	Listening: 36 (27.1%)	EIKEN: 51 (38.3%)	Japanese Private School: 22 (16.5%)	Bachelor's Degree: 20 (15%)	Listening: 17 (12.8%)	Listening: 15 (11.3%)	English lessons could be improved: 20 (15%)
	Reading: 73 (54.9%)	Reading: 12 (9%)	TOEIC: 51 (38.3%)	Japanese University: 19 (14.3%)	Master's Degree: 2 (1.5%)	Reading: 8 (6%)	Reading: 13 (9.8%)	Want more opportunities for English usage: 23 (17.3%)
	Speaking: 11 (8.3%)	Speaking: 69 (51.9%)	TOEFL: 28 (21.1%)	Foreign Public School: 0 (0%)	Doctorate Degree: 0 (0%)	Speaking: 38 (28.6%)	Speaking: 29 (21.8%)	Want to improve English skills: 20 (15%)
	Writing: 14 (10.5%)	Writing: 20 (15%)	IELTS: 8 (6%)	Foreign Private School: 0 (0%)		Writing: 0 (0%)	Writing: 5 (3.8%)	Others: 10 (7.5%)
	All skills: 2 (1.5%)	Everything: 55 (41.4%)	EPEMP: 0 (0%)	Foreign University: 2 (1.5%)		Everything: 36 (27.1%)	Everything: 65 (48.9%)	
			OET: 0 (0%)					
First year	Nothing: 3 (15.8%)	Nothing: 0 (0%)	Nothing: 8 (42.1%)	Japanese Public School: 15 (78.9%)	None: 18 (94.7%)	Nothing: 6 (31.6%)	Nothing: 1 (5.3%)	No comments: 10 (52.6%)
	Listening: 7 (36.8%)	Listening: 4 (21.1%)	EIKEN: 9 (47.4%)	Japanese Private School: 3 (15.8%)	Bachelor's Degree: 1 (5.3%)	Listening: 1 (5.3%)	Listening: 2 (10.5%)	English lessons could be improved: 3 (15.8%)
	Reading: 12 (63.2%)	Reading: 3 (15.8%)	TOEIC: 4 (21.1%)	Japanese University: 1 (5.3%)	Master's Degree: 0 (0%)	Reading: 0 (0%)	Reading: 0 (0%)	Want more opportunities for English usage: 2 (10.5%)
	Speaking: 3 (15.8%)	Speaking: 12 (63.2%)	TOEFL: 4 (21.1%)	Foreign Public School: 0 (0%)	Doctorate Degree: 0 (0%)	Speaking: 7 (36.8%)	Speaking: 5 (26.3%)	Want to improve English skills: 3 (15.8%)
	Writing: 2 (10.5%)	Writing: 8 (42.1%)	IELTS: 2 (10.5%)	Foreign Private School: 0 (0%)		Writing: 0 (0%)	Writing: 1 (5.3%)	Others: 1 (5.3%)
	All Skills: 0 (0%)	Everything: 6 (31.6%)	EPEMP: 0 (0%)	Foreign University: 0 (0%)		Everything: 5 (26.3%)	Everything: 10 (52.6%)	
			OET: 0 (0%)					
Second year	Nothing: 2 (22.2%)	Nothing: 0 (0%)	Nothing: 4 (44.4%)	Japanese Public School: 6 (66.7%)	None: 8 (88.9%)	Nothing: 2 (22.2%)	Nothing: 0 (0%)	No comments: 4 (44.4%)
	Listening: 5 (55.6%)	Listening: 4 (44.4%)	EIKEN: 2 (22.2%)	Japanese Private School: 2 (22.2%)	Bachelor's Degree: 1 (11.1%)	Listening: 0 (0%)	Listening: 1 (11.1%)	English lessons could be improved: 1 (11.1%)
	Reading: 5 (55.6%)	Reading: 1 (11.1%)	TOEIC: 3 (33.3%)	Japanese University: 1 (11.1%)	Master's Degree: 0 (0%)	Reading: 1 (11.1%)	Reading: 1 (11.1%)	Want more opportunities for English usage: 2 (22.2%)
	Speaking: 0 (0%)	Speaking: 7 (77.8%)	TOEFL: 2 (22.2%)	Foreign Public School: 0 (0%)	Doctorate Degree: 0 (0%)	Speaking: 3 (33.3%)	Speaking: 2 (22.2%)	Want to improve English skills: 2 (22.2%)
	Writing: 2 (22.2%)	Writing: 2 (22.2%)	IELTS: 0 (0%)	Foreign Private School: 0 (0%)		Writing: 0 (0%)	Writing: 1 (11.1%)	Others: 0 (0%)
	All skills: 0 (0%)	Everything: 2 (22.2%)	EPEMP: 0 (0%)	Foreign University: 0 (0%)		Everything: 3 (33.3%)	Everything: 4 (44.4%)	
			OET: 0 (0%)					
Third year	Nothing: 9 (25%)	Nothing: 0 (0%)	Nothing: 10 (27.8%)	Japanese Public School: 22 (61.1%)	None: 27 (75%)	Nothing: 6 (16.7%)	Nothing: 2 (5.6%)	No comments: 11 (30.6%)
	Listening: 7 (19.4%)	Listening: 10 (27.8%)	EIKEN: 15 (41.7%)	Japanese Private School: 6 (16.7%)	Bachelor's Degree: 8 (22.2%)	Listening: 8 (22.2%)	Listening: 5 (13.9%)	English lessons could be improved: 6 (16.7%)
	Reading: 20 (55.6%)	Reading: 2 (5.6%)	TOEIC: 16 (44.4%)	Japanese University: 7 (19.4%)	Master's Degree: 1 (2.8%)	Reading: 4 (11.1%)	Reading: 0 (0%)	Want more opportunities for English usage: 10 (27.8%)
	Speaking: 3 (8.3%)	Speaking: 14 (38.9%)	TOEFL: 10 (27.8%)	Foreign Public School: 0 (0%)	Doctorate Degree: 0 (0%)	Speaking: 10 (27.8%)	Speaking: 11 (30.6%)	Want to improve English skills: 4 (11.1%)
	Writing: 5 (13.9%)	Writing: 3 (8.3%)	IELTS: 4 (11.1%)	Foreign Private School: 0 (0%)		Writing: 0 (0%)	Writing: 1 (2.8%)	Others: 4 (11.1%)
	All Skills: 1 (2.8%)	Everything: 20 (55.6%)	EPEMP: 0 (0%)	Foreign University: 1 (2.8%)		Everything: 8 (22.2%)	Everything: 17 (47.2%)	
			OET: 0 (0%)					
Fourth year	Nothing: 8 (21.6%)	Nothing: 0 (0%)	Nothing: 13 (35.1%)	Japanese Public School: 26 (70.3%)	None: 31 (83.8%)	Nothing: 8 (21.6%)	Nothing: 0 (0%)	No comments: 17 (45.9%)
	Listening: 16 (43.2%)	Listening: 8 (21.6%)	EIKEN: 16 (43.2%)	Japanese Private School: 5 (13.5%)	Bachelor's Degree: 5 (13.5%)	Listening: 2 (5.4%)	Listening: 4 (10.8%)	English lessons could be improved: 7 (18.9%)
	Reading: 22 (59.5%)	Reading: 3 (8.1%)	TOEIC: 16 (43.2%)	Japanese University: 6 (16.2%)	Master's Degree: 1 (2.7%)	Reading: 2 (5.4%)	Reading: 5 (13.5%)	Want more opportunities for English usage: 6 (16.2%)
	Speaking: 3 (8.1%)	Speaking: 20 (54.1%)	TOEFL: 6 (16.2%)	Foreign Public School: 0 (0%)	Doctorate Degree: 0 (0%)	Speaking: 11 (29.7%)	Speaking: 5 (13.5%)	Want to improve English skills: 4 (10.8%)
	Writing: 2 (5.4%)	Writing: 5 (13.5%)	IELTS: 1 (2.7%)	Foreign Private School: 0 (0%)		Writing: 0 (0%)	Writing: 2 (5.4%)	Others: 3 (8.1%)
	All Skills: 1 (2.7%)	Everything: 16 (43.2%)	EPEMP: 0 (0%)	Foreign University: 0 (0%)		Everything: 14 (37.8%)	Everything: 21 (56.8%)	
			OET: 0 (0%)					
Fifth year	Nothing: 11 (45.8%)	Nothing: 0 (0%)	Nothing: 5 (20.8%)	Japanese Public School: 15 (62.5%)	None: 20 (83.3%)	Nothing: 8 (33.3%)	Nothing: 2 (8.3%)	No comments: 13 (54.2%)
	Listening: 7 (29.2%)	Listening: 7 (29.2%)	EIKEN: 8 (33.3%)	Japanese Private School: 5 (20.8%)	Bachelor's Degree: 4 (16.7%)	Listening: 4 (16.7%)	Listening: 2 (8.3%)	English lessons could be improved: 2 (8.3%)
	Reading: 9 (37.5%)	Reading: 1 (4.2%)	TOEIC: 9 (37.5%)	Japanese University: 3 (12.5%)	Master's Degree: 0 (0%)	Reading: 0 (0%)	Reading: 6 (25%)	Want more opportunities for English usage: 2 (8.3%)
	Speaking: 2 (8.3%)	Speaking: 11 (45.8%)	TOEFL: 5 (20.8%)	Foreign Public School: 0 (0%)	Doctorate Degree: 0 (0%)	Speaking: 7 (29.2%)	Speaking: 2 (8.3%)	Want to improve English skills: 6 (25%)
	Writing: 3 (12.5%)	Writing: 2 (8.3%)	IELTS: 1 (4.2%)	Foreign Private School: 0 (0%)		Writing: 0 (0%)	Writing: 0 (0%)	Others: 1 (4.2%)
	All Skills: 0 (0%)	Everything: 10 (41.7%)	EPEMP: 0 (0%)	Foreign University: 1 (4.2%)		Everything: 5 (20.8%)	Everything: 12 (50%)	
			OET: 0 (0%)					
Sixth year	Nothing: 3 (37.5%)	Nothing: 0 (0%)	Nothing: 3 (37.5%)	Japanese Public School: 6 (75%)	None: 7 (87.5%)	Nothing: 4 (50%)	Nothing: 1 (12.5%)	No comments: 4 (50%)
	Listening: 0 (0%)	Listening: 3 (37.5%)	EIKEN: 1 (12.5%)	Japanese Private School: 1 (12.5%)	Bachelor's Degree: 1 (12.5%)	Listening: 2 (25%)	Listening: 1 (12.5%)	English lessons could be improved: 1 (12.5%)
	Reading: 5 (62.5%)	Reading: 2 (25%)	TOEIC: 3 (37.5%)	Japanese University: 1 (12.5%)	Master's Degree: 0 (0%)	Reading: 1 (12.5%)	Reading: 1 (12.5%)	Want more opportunities for English usage: 1 (12.5%)
	Speaking: 0 (0%)	Speaking: 5 (62.5%)	TOEFL: 1 (12.5%)	Foreign Public School: 0 (0%)	Doctorate Degree: 0 (0%)	Speaking: 0 (0%)	Speaking: 4 (50%)	Want to improve English skills: 1 (12.5%)
	Writing: 0 (0%)	Writing: 0 (0%)	IELTS: 0 (0%)	Foreign Private School: 0 (0%)		Writing: 0 (0%)	Writing: 0 (0%)	Others: 1 (12.5%)
	All skills: 0 (0%)	Everything: 1 (12.5%)	EPEMP: 0 (0%)	Foreign University: 0 (0%)		Everything: 1 (12.5%)	Everything: 1 (12.5%)	
			OET: 0 (0%)					
Males	Nothing: 18 (28.1%)	Nothing: 0 (0%)	Nothing: 24 (37.5%)	Japanese Public School: 44 (68.8%)	None: 53 (82.8%)	Nothing: 14 (21.9%)	Nothing: 2 (3.1%)	No comments: 30 (46.9%)
	Listening: 13 (20.3%)	Listening: 21 (32.8%)	EIKEN: 23 (35.9%)	Japanese Private School: 11 (17.2%)	Bachelor's Degree: 10 (15.6%)	Listening: 10 (15.6%)	Listening: 8 (12.5%)	English lessons could be improved: 11 (17.2%)
	Reading: 39 (60.9%)	Reading: 8 (12.5%)	TOEIC: 20 (31.3%)	Japanese University: 9 (14.1%)	Master's Degree: 1 (1.6%)	Reading: 6 (9.4%)	Reading: 9 (14.1%)	Want more opportunities for English usage: 7 (10.9%)
	Speaking: 4 (6.3%)	Speaking: 33 (51.6%)	TOEFL: 10 (15.6%)	Foreign Public School: 0 (0%)	Doctorate Degree: 0 (0%)	Speaking: 22 (34.4%)	Speaking: 15 (23.4%)	Want to improve English skills: 9 (14.1%)
	Writing: 8 (12.5%)	Writing: 9 (14.1%)	IELTS: 3 (4.7%)	Foreign Private School: 0 (0%)		Writing: 0 (0%)	Writing: 4 (6.3%)	Others: 6 (9.4%)
	All skills: 2 (3.1%)	Everything: 24 (37.5%)	EPEMP: 0 (0%)	Foreign University: 0 (0%)		Everything: 12 (18.8%)	Everything: 26 (40.6%)	
			OET: 0 (0%)					
Females	Nothing: 18 (26.1%)	Nothing: 0 (0%)	Nothing: 19 (27.5%)	Japanese Public School: 46 (66.7%)	None: 58 (84.1%)	Nothing: 20 (29%)	Nothing: 4 (5.8%)	No comments: 29 (42%)
	Listening: 29 (42%)	Listening: 15 (21.7%)	EIKEN: 28 (40.6%)	Japanese Private School: 11 (15.9%)	Bachelor's Degree: 10 (14.5%)	Listening: 7 (10.1%)	Listening: 7 (10.1%)	English lessons could be improved: 9 (13%)
	Reading: 34 (49.3%)	Reading: 4 (5.8%)	TOEIC: 31 (44.9%)	Japanese University: 10 (14.5%)	Master's Degree: 1 (1.4%)	Reading: 2 (2.9%)	Reading: 4 (5.8%)	Want more opportunities for English usage: 16 (23.2%)
	Speaking: 6 (8.7%)	Speaking: 36 (52.2%)	TOEFL: 18 (26.1%)	Foreign Public School: 0 (0%)	Doctorate Degree: 0 (0%)	Speaking: 16 (23.2%)	Speaking: 14 (20.3%)	Want to improve English skills: 11 (15.9%)
	Writing: 6 (8.7%)	Writing: 11 (15.9%)	IELTS: 5 (7.2%)	Foreign Private School: 0 (0%)		Writing: 0 (0%)	Writing: 1 (1.4%)	Others: 4 (5.8%)
	All skills: 0 (0%)	Everything: 31 (44.9%)	EPEMP: 0 (0%)	Foreign University: 2 (2.9%)		Everything: 24 (34.8%)	Everything: 39 (56.5%)	
			OET: 0 (0%)					

## Discussion

It is clear that a considerable number of Japanese medical students may be uninterested in English and Medical English education based on the fact that only a minority answered the questionnaire. That does not necessarily indicate disinterest in the remaining enrolled students, as some may have been busy or away during the three weeks when the study was conducted; however, beyond speculation, it is impossible to determine all the reasons behind why questionnaires were missed by such a large number of students. Based on stratified response rates, interest appears to peak in the third and fourth years before dropping back down in the fifth and sixth years. Nevertheless, it would be wise to include an incentive for participants when conducting future studies of a similar nature in order to improve response rates. For example, a monetary gift or raffle entry for a valuable item, among others, may push more students to participate. The current results would be open to debate, since the sample may not be truly representative of all students at the medical school. Additionally, surveying students in other cities, especially those in urban settings, may reveal significant differences in responses.

This pilot study revealed that self-reported proficiency and confidence ratings are low among many Japanese medical students. The majority rated themselves as having no proficiency or elementary proficiency in both English and Medical English. In fact, only two out of 133 participants believed they had full or native proficiency. Unfortunately, low ratings were observed in both junior and senior students at a similar proportion, which supports the notion that language proficiency does not significantly improve with the enrollment year. Ideally, medical schools should re-administer the questionnaire to the same group of students over the course of six years in order to make this conclusion more reliable. Similar responses were noted in levels of confidence and English class understanding among all medical students, which further supports the proficiency findings. Confidence in reading and listening skills was significantly higher than in speaking and writing skills, which is likely a result of the current educational policies at public middle schools and high schools. English education at the school level focuses primarily on reading and listening skills in a typical didactic setting to prepare students for university entrance examinations, which rely heavily on these English components [24].

Responses showed that the majority of students start their first year of medical school directly after graduating from a public high school in Japan, without pursuing higher degrees, and would therefore possess equivalent levels of English skills unless a significant personal effort was made by some towards improving their own proficiency. Unfortunately, most students would not have had any kind of incentive to pursue English language education, beyond the compulsory teaching at schools within what is defined by the general system and national curriculum [24]. The EIKEN and TOEIC exams were much more commonly taken compared to others, owing to their popularity, benefits, and access within Japan. However, the fraction of medical students sitting for these exams remains low, making it impossible to use them for large-scale proficiency determination. Attendance of clinical sessions in English was noted to be relatively higher in junior years, which could be explained by the introduction of new classes and the continued expansion of Medical English and English education at the university. However, this did not translate into more opportunities for medical students to present in English, which remained limited to one to three times throughout the six years at medical school. Interestingly, some participants reported presenting ten times during the course of their medical education, thereby indicating a non-uniform experience in some instances.

The question regarding lifetime duration for studying English seemed to be understood in one of two ways. Some students included the mandatory school years in their calculations, while others left them out. In future versions of the questionnaire, it is imperative to clarify if those years should be taken into account or not by appropriately modifying the question or providing a side note. Weekly use of the English language in personal settings was quite low, but even lower in professional settings. This was in fact expected because of societal and cultural reasons, where usage of the native Japanese language is the norm [24]. Despite that, most respondents seemed to acknowledge the importance of Medical English for their careers. About half of the participants indicated that being proficient in all English skills was important for professional progression, while a quarter of them highlighted speaking as the most important skill for both personal and career-related uses. Consequently, all participating medical students indicated their intention to improve their English and Medical English proficiency. Half of these students primarily hoped to improve their speaking skills, but a considerable proportion wanted to build upon all of their English language skills. The only open-ended question yielded suggestions about improving classes and providing an opportunity for the regular use of English and Medical English. For the only two students with high levels of self-reported proficiency, both had studied English for over 15 years and had attended over 50 medical classes in English within a single year.

Potential methods for improving Medical English proficiency

Various propositions have been made by several experts involved in the advancement of English proficiency in Japan. Some have suggested improving English education at the school level by updating classes, improving interactions, enhancing teaching methods, and revising curriculums to include speaking and writing components [24,25]. However, many have conceded that any real gains in proficiency cannot be attained by modifying teaching methods alone, but would rather require some level of cultural awareness, personality changes, and societal adoption [24,26-28]. A few of the students involved in this study provided some positive comments on what they would likely find beneficial for English and Medical English advancement within Japanese medical schools. Many of them hoped for more opportunities to practice and use English in a professional setting, while others wished to see improvements in teaching and classes. Identical suggestions were made previously by a different group of students who were asked similar questions more than a decade prior to this survey [21]. Since then, many medical schools have been able to introduce and advance Medical English courses within their curricula [16,17,19,20]. Unfortunately, no significant gains were made in terms of opportunities for English use and cultural or societal changes, at least in the institution under investigation.

Ideally, the Japanese government and population would adopt some of the suggested educational and societal policy changes needed for advancing English among students to allow for a gradual improvement in language usage and proficiency. Realistically, a large resistance to enacting major changes will continue to exist for the foreseeable future. Multiple theories and reasons have been given as to why such resistance exists, but this topic is beyond the scope of the current study. Based on the results obtained from this pilot survey, it is clear that once the majority of Japanese medical students begin their studies at medical schools, their low English language proficiency makes it difficult to rely solely on the university’s English classes to improve their Medical English skills. Consequently, self-rated confidence and proficiency remain low throughout enrollment. For that reason, many would have to utilize extracurricular lessons and exert considerable personal effort in order to achieve an adequate level of proficiency. At this point in time, it would seem impossible for medical schools to provide students with a significantly larger number of Medical English classes and interactions without sacrificing critical Japanese medical courses, required for licensing preparation and medical practice in Japan.

While it is crucial to continue providing and improving Medical English courses and opportunities for English usage at Japanese medical schools, we believe that this alone is unlikely to greatly affect levels of proficiency among medical students without employing methods that allow for regular weekly interactions and English practice. Students may opt to find their own ways for consistent use of and exposure to English, such as by making English-speaking friends, watching English movies, or playing games in English. Private lessons would also allow for consistent usage of English, despite being non-medical in nature. Another possible way would be for medical schools to sponsor or help create extra-curricular Medical English clubs that would hold sessions on a regular weekly basis. These would potentially enable medical students to practice English and Medical English in a non-formal quasi-professional setting. Session content may vary depending on the proficiency levels of medical students, but may include interactive clinical student-centered approaches like problem-based learning (PBL), objective structured clinical examination (OSCE), viva voce, journal club, and others. The main disadvantage would be requiring time outside a student’s regular schedule, and the difficulty in enabling attendance of most medical students in a single enrollment year, especially if this initiative is optional. These clubs could potentially be made available to students based on individual needs through an organized and structured approach, which would undoubtedly improve English and Medical English confidence and proficiency levels.

Limitations

Some of the limitations associated with this pilot study were: the small percentage of participants and high number of non-responders, the possibility of sampling bias due to the inclusion of students living exclusively in a rural setting, the geographic limitation of the study within Japan that may not necessarily represent other areas within the country, the potential for recall and response bias, the social desirability and extreme responding bias involved in the usage of Likert scales, the lack of prior validation, especially in questions related to self-rated proficiency and language skills. These limitations are expected given the nature of the study and its pilot status.

## Conclusions

The proposed questionnaire was able to give valuable information about language skills and proficiency levels, but would require an incentive for improved participation. The pilot analysis showed that English and Medical English proficiency levels remain low with limited opportunities for using English in some areas within Japan. It may be beneficial to provide Japanese medical students with more occasions where they can use or practice their English skills.
